# Tumour-associated macrophages enhance breast cancer malignancy via inducing ZEB1-mediated DNMT1 transcriptional activation

**DOI:** 10.1186/s13578-022-00913-4

**Published:** 2022-10-22

**Authors:** Zhongwei Li, Pengfei Wang, Wenjie Cui, Hongmei Yong, Diandian Wang, Tiesuo Zhao, Wenwen Wang, Ming Shi, Junnian Zheng, Jin Bai

**Affiliations:** 1 Cancer Institute, Xuzhou Medical University, 84 West Huaihai Road, Xuzhou, 221002 Jiangsu China; 2 Center of Clinical Oncology, the Affiliated Hospital of Xuzhou Medical University, Xuzhou, Jiangsu China; 3 Jiangsu Center for the Collaboration and Innovation of Cancer Biotherapy, Cancer Institute, Xuzhou Medical University, 84 West Huaihai Road, Xuzhou, 221002 Jiangsu China; 4 Department of Respiratory and Critical Care Medicine, The Municipal Hospital, Affiliated to Xuzhou Medical University, Xuzhou, Jiangsu China; 5 Intensive Care Unit, The Second Affiliated Hospital of Xuzhou Medical University, Xuzhou, Jiangsu, China; 6 Department of Immunology, School of Basic Medical Sciences, Xinxiang Medical University, Xinxiang, Henan China

**Keywords:** DNMT1, ZEB1, Tumour-associated macrophages, Metastasis, Breast cancer

## Abstract

**Background:**

DNMT1 has been shown to be highly expressed in a variety of cancers, including breast cancer. However, the mechanism is not very clear. Therefore, we aim to reveal the mechanism of DNMT1 highly express in breast cancer. And we also want to explore the role of DNMT1 in tumour microenvironment promoting breast cancer progression.

**Results:**

In this study, we demonstrate that DNMT1 is overexpressed in breast cancer and that DNMT1 promotes breast cancer tumorigenesis and metastasis. We discovered that ZEB1 activates DNMT1 expression in breast cancer cells by recruiting P300 binding to the DNMT1 promoter and increasing its acetylation. Moreover, we revealed that tumour-associated macrophages (TAMs) increase DNMT1 expression in breast cancer cells via the IL-6-pSTAT3-ZEB1-DNMT1 axis in the tumour microenvironment. DNMT1 is required for TAM-mediated breast cancer cell migration. In addition, we confirmed that there were positive correlations among CD163 (TAM marker) expression, ZEB1 expression and DNMT1 expression in breast cancer patient tissues.

**Conclusions:**

Our study indicates that DNMT1 is necessary for TAM-mediated breast cancer metastasis. Decitabine (DAC), as a specific DNA methylation inhibitor and FDA-approved drug, is a bona fide drug for breast cancer treatment.

**Supplementary Information:**

The online version contains supplementary material available at 10.1186/s13578-022-00913-4.

## Background

DNMT1, as an important epigenetic enzyme, regulates the expression of various genes by catalysing DNA methylation [1]. Studies have shown that DNMT1 is highly expressed in many cancers. DNMT1 has many functions in cancer progression [2]. For instance, our and other reports have demonstrated that DNMT1 is able to promote prostate cancer progression by increasing EZH2 expression [3, 4]. Moreover, studies also revealed that inhibition of DNMT1 expression can attenuate cancer proliferation and motility [5, 6]. The DNA methylation inhibitor decitabine (DAC), an FDA-approved drug, has been proven to suppress prostate cancer tumorigenesis [4, 7–9]. However, the detailed mechanism of high DNMT1 expression in cancers has not been clear until now.

The tumour microenvironment (TME) plays a key role in cancer progression, especially for promoting cancer metastasis [10, 11]. The TME contains various types of immune cells, such as tumour-associated macrophages (TAMs), tumour-associated fibroblast cells, NK cells and DCs [12, 13]. Many reports have shown that TAMs can facilitate breast cancer cell metastasis. For instance, our previous study demonstrated that TAMs enhance breast cancer cell distant metastasis by strengthening EZH2 protein stability [14]. However, whether DNMT1 plays a key role in TAM-induced breast cancer progression has not yet been reported.

ZEB1 plays a critical role in facilitating tumorigenesis and cancer cell distant metastasis [15]. ZEB1, as a major EMT (Epithelial-Mesenchymal Transition) inducer, can promote cancer cell migration and metastasis by repressing the expression of its target gene E-cadherin [16, 17]. Many studies have shown that ZEB1 suppresses its target gene expression by binding to its target gene promoter region and inhibiting its transcription [15, 17]. Nevertheless, a few reports have revealed that ZEB1 can activate target gene transcription [18–20]. For instance, it has been shown that ZEB1 interacting with P300 can activate target gene transcription by increasing target gene promoter histone acetylation [21, 22]. Whether ZEB1 can promote breast cancer progression by activating its target gene transcription deserves to be explored.

In this study, we demonstrate that DNMT1 is highly expressed and promotes breast cancer cell proliferation and migration in vitro and in vivo. We found that ZEB1 can directly activate DNMT1 transcription. Moreover, we disclose that ZEB1 interacts with P300 and that ZEB1-P300 is enriched on the DNMT1 promoter, which leads to an increase in DNMT1 promoter histone acetylation and subsequent transcriptional activation. In addition, we revealed that TAM-stimulated DNMT1 transcriptional expression via the IL-6-pSTAT3-ZEB1-DNMT1 axis is necessary for TAMs to promote breast cancer metastasis in the tumour microenvironment. Finally, we confirmed that there were positive correlations between CD163 (TAM marker) and ZEB1 expression, CD163 and DNMT1 expression, and ZEB1 and DNMT1 expression. Our study indicates that DNMT1 is critical for TAM-mediated breast cancer metastasis. DAC, as a DNMT1-specific inhibitor and FDA-approved drug, is a promising drug for breast cancer treatment.

## Results

### Ectopic expression of DNMT1 facilitates breast cancer cell proliferation

**Fig. 1:**
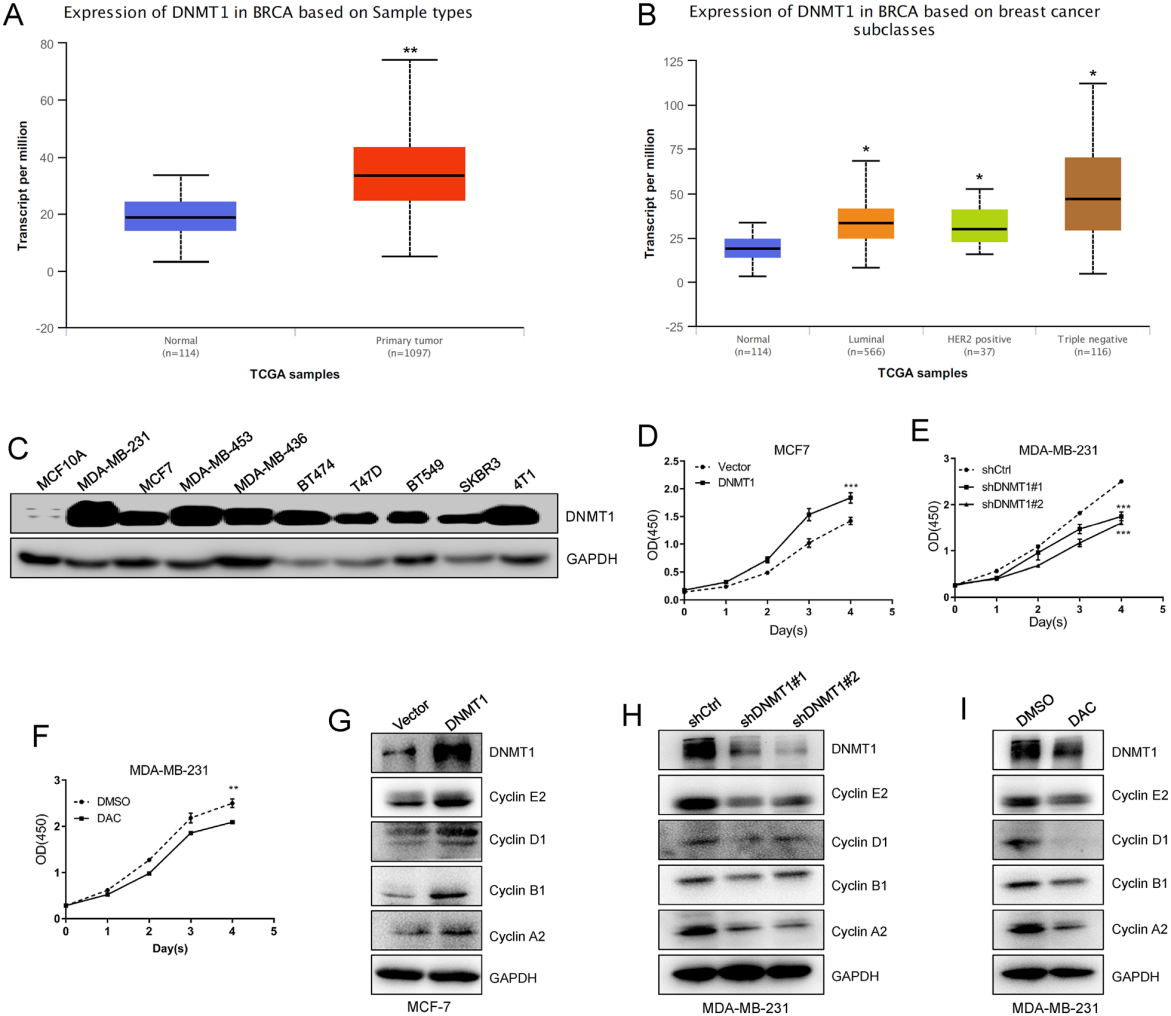
DNMT1 enhances breast cancer cells proliferation in vitro. **A** Analysis of DNMT1 expression in normal tissues and primary breast cancer tissues in TCGA database. **B** Analysis of DNMT1 expression in different subtypes of breast cancer tissues. **C** Detection of DNMT1 expression in breast cancer cell lines and MCF10A mammary epithelial cell line. **D** Assessment of MCF7-Vector cells and MCF7-DNMT1 cells proliferation abilities by CCK-8 assays. **E**, **F** CCK-8 analysis of MDA-MB-231 cells proliferation ability after knockdown DNMT1 or treated with DAC. **G** Western blots detected DNMT1, Cyclin-E2, Cyclin-D1, Cyclin-B1 and Cyclin-A2 expression in MCF7-Vector cells and MCF7-DNMT1 cells. **H**, **I** Detection of DNMT1, Cyclin-E2, Cyclin-D1, Cyclin-B1 and Cyclin-A2 expression by immune blots after depletion of DNMT1 or treated with DAC in MDA-MB-231 cells. Data are represented as mean ± SD of three or four independent experiments, and *p < 0.05, **p < 0.01, ***p < 0.001 (Student’s *t* test)

We wanted to explore the potential epigenetic driver genes that enhance breast cancer progression. A variety of reports have demonstrated that methylations of DNA, RNA and protein have a great effect on tumorigenesis and cancer metastasis. First, we analysed the expression of several key DNA methyltransferases, RNA methyltransferases and protein methyltransferases in the TCGA database. The results showed that the DNA methyltransferase DNMT1 was dramatically overexpressed in breast cancer tissues compared with normal breast tissues (Fig. 1A). We consistently found that DNMT1 was highly expressed in luminal subtype, HER2 positive subtype and triple negative breast cancer tissues (Fig. 1B). Then, our western blot results also revealed that DNMT1 is highly expressed in various breast cancer cell lines (MDA-MB-231, MCF7, MDA-MB-453, etc.) compared with that in MCF10A cells (mammary epithelial cells) (Fig. 1C). This means that DNMT1 is highly expressed both in breast cancer cell lines and in breast cancer tissues. This finding indicates that DNMT1 may play a key role in breast cancer progression.

Subsequently, we found that ectopic expression of DNMT1 promotes MCF7 breast cancer cell proliferation, while knockdown of DNMT1 inhibits MCF7 breast cancer cell proliferation (Fig. 1D and E). In addition, we revealed that the proliferation ability of MCF7 cells was significantly decreased after treatment with the DNA methylation-specific inhibitor DAC (Fig. 1F). Finally, we showed that cell cyclin-associated proteins (Cyclin A2, Cyclin B1, Cyclin D1 and Cyclin E1) were increased after overexpression of DNMT1 in MCF7-DNMT1 cells compared with MCF7-Vector cells (Fig. 1G). However, these cell cyclin markers were decreased after silencing DNMT1 expression or treatment with DAC in MCF7 cells (Fig. 1H and I. Overall, our data show that DNMT1 is able to promote breast cancer cell proliferation.

### DNMT1 induces the EMT program and promotes cell motility in breast cancer

**Fig. 2:**
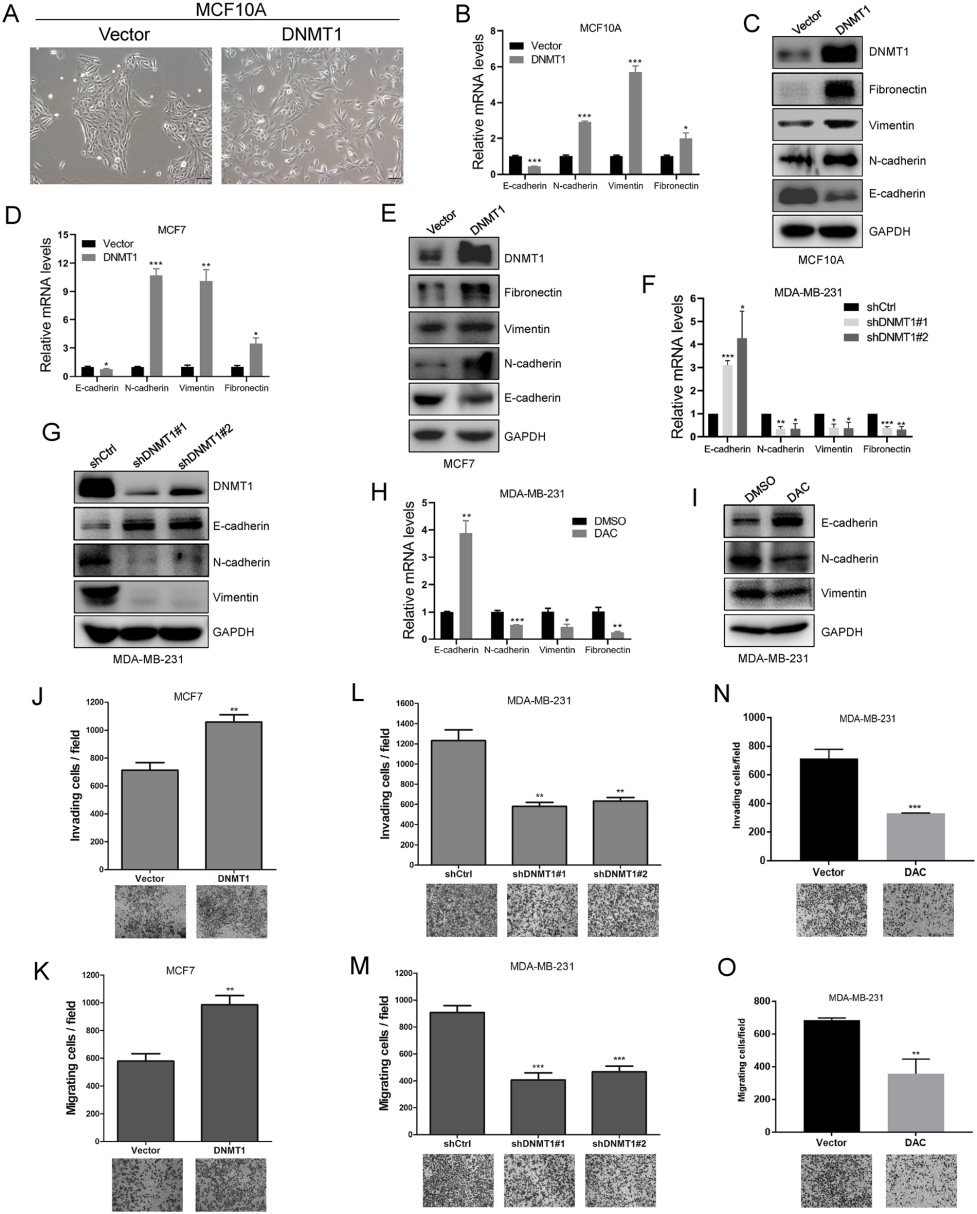
DNMT1 promotes EMT progression and facilitates cells motility in breast cancer. **A** The morphological change of MCF10A cells was examined by phase constrast microscopy after overexpressing DNMT1. Scale bar: 100 μm. **B**–**E** Detection of EMT markers expression in mRNA level and protein level after overexpression of DNMT1 in MCF10A cells and MCF7 cells. **F**–**I** qRT-PCR assays and western blot assays analyzed EMT markers expression cells after knockdown DNMT1 or treated with DAC in MDA-MB-231 cells. **J–O** Assessment of cells motility by invasion assays (**J**, **L**, **N**) and migration assays (**K**, **M**, **O**) in MCF7-Vector/DNMT1 cells, MDA-MB-231-shCtrl/shDNMT1 cells, or MDA-MB-231 cells treated with DMSO/DAC. Data are represented as mean ± SD of three or four independent experiments, and *p < 0.05, **p < 0.01, ***p < 0.001 (Student’s *t* test)

We next explored whether DNMT1 can affect breast cancer cell motility. We revealed that MCF10A-DNMT1 cells underwent a change into the spindle-shaped, fibroblastic-like phenotype, with little cell–cell contact (Fig. 2A). We also discovered that MCF10A-DNMT1 and MCF7-DNMT1 cells exhibited a decrease in the epithelial marker E-cadherin, accompanied by an increase in the mesenchymal markers N-cadherin, Vimentin and Fibronectin at the mRNA and protein levels (Fig. 2B–E). Meanwhile, we found that E-cadherin was increased, while N-cadherin, Vimentin and Fibronectin were decreased at the transcriptional level and protein level in MDA-MB-231-shDNMT1 breast cancer cells (Fig. 2F, G). We observed similar results in MDA-MB-231 cells after treatment with DAC (Fig. 2H, I ). Our data strongly show that DNMT1 can induce the EMT program in breast cancer cells. This finding suggests that DNMT1 may play a critical role in breast cancer cell migration.

Moreover, we detected DNMT1 functions in breast cancer cell motility by transwell migration assays and Matrigel invasion assays. As our results showed, the migration and invasion abilities were elevated when DNMT1 was overexpressed in nonmetastatic MCF7 breast cancer cells (Fig. 2J, K). In contrast, we also discovered that knockdown of DNMT1 strongly suppressed breast cancer cell migratory and invasive behaviours in invasive MDA-MB-231 cells (Fig. 2L, M). In addition, we showed that the DNA methylation-specific inhibitor DAC could significantly attenuate MDA-MB-231 cell motility (Fig. 2N, O). Taken together, our results indicate that DNMT1 is required for breast cancer cell EMT progression. DNMT1 is necessary for breast cancer cell migration and invasion.

### DNMT1 knockdown inhibits breast cancer tumorigenesis and metastasis in vivo

**Fig. 3:**
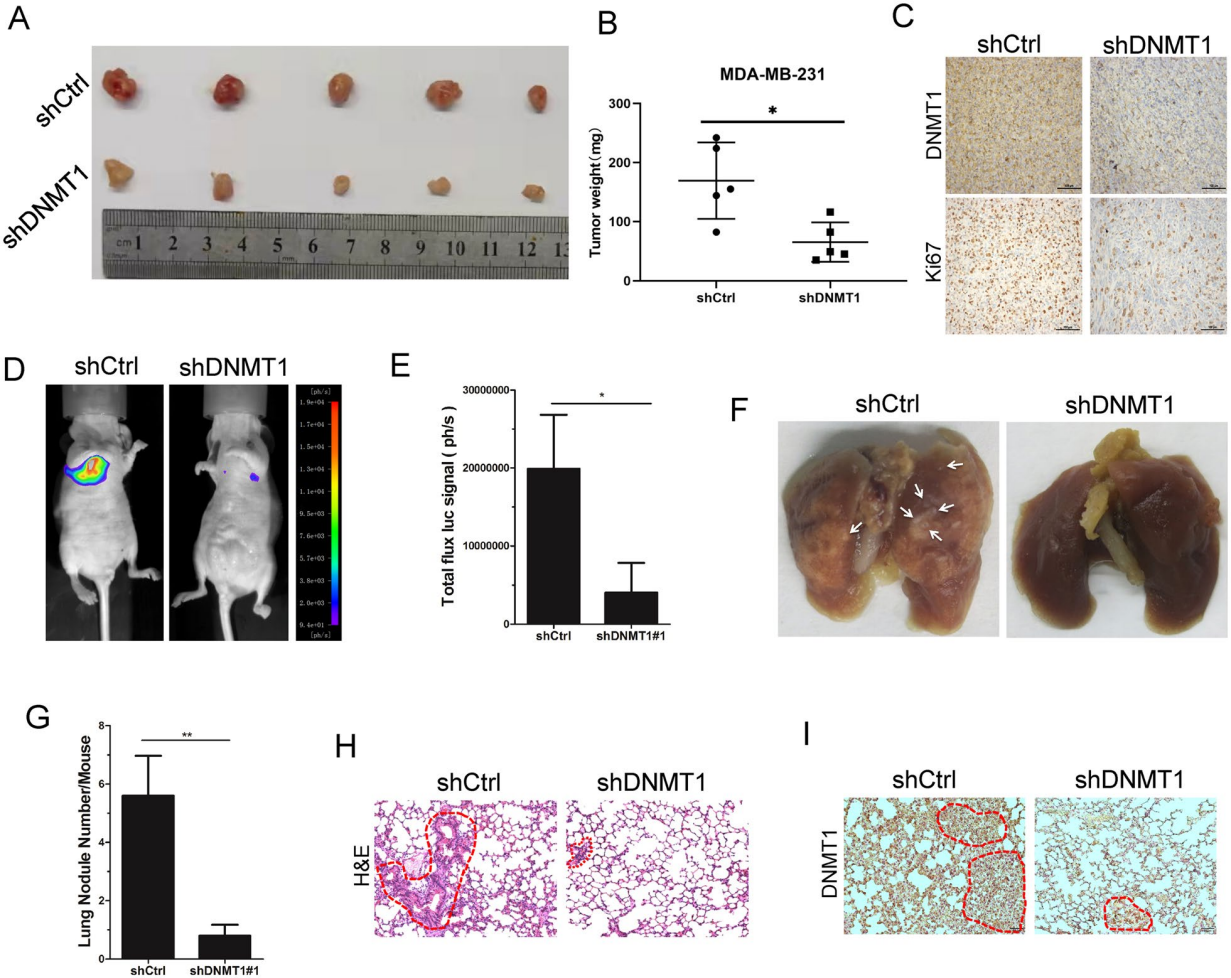
DNMT1 facilitates breast cancer cells tumorigenesis and metastasis in vivo. **A**, **B** MDA-MB-231-shCtrl/shDNMT1#1 cells were subcutaneously injected into the female nude mice, respectively. n = 5 for each group. After 4 weeks the xenograft tumours were detached (**A**). Effect of knockdown DNMT1 on the xenograft model was assessed by evaluating tumour weight (**B**). **C** IHC staining detected Ki67 and DNMT1 expression in xenograft tumours. **D**, **E** Representative bioluminescence images of lung metastasis (**D**) in mice injected with MDA-MB-231-shCtrl or MDA-MB-231-shDNMT1 cells via tail veins, and the metastasis were quantified by measuring the photo flux (**E**). **F**–**H** After 8 weeks, nude mice were sacrificed and lung metastatic nodules (**F**) were examined macroscopically (**G**) or detected by H&E staining (**H**). The white arrows denote the metastatic nodules. **I** Representative images of the IHC staining of DNMT1 in nude mice lung metastasis sections. Scale bars: 100 μm. Data are represented as mean ± SD of three or four independent experiments, and *p < 0.05, **p < 0.01, ***p < 0.001 (Student’s *t* test)

The above data confirmed that DNMT1 is able to facilitate breast cancer cell proliferation and motility in vitro. Next, we explored the functions of DNMT1 in breast cancer tumorigenesis and metastasis in vivo*.* First, MDA-MB-231-shCtrl cells or MDA-MB-231-shDNMT1 cells were subcutaneously injected into BALB/c female nude mice. We detected the formed-tumour sizes in the control group and DNMT1 silencing group when the mice were sacrificed 4 weeks later. Our results showed that the tumours in the DNMT1 knockdown group were much smaller than those in the control group (Fig. 3A). We also confirmed that the average tumour weight in the DNMT1 knockdown group was lower than that in the control group (Fig. 3B). In addition, we revealed that the expression of Ki67 (an important proliferation marker) was decreased in MDA-MB-231-shDNMT1-formed tumours compared with in MDA-MB-231-shCtrl-formed tumours (Fig. 3C). These results indicate that DNMT1 can promote breast cancer tumorigenesis in vivo.

Moreover, to evaluate whether DNMT1 can affect breast cancer metastasis in vivo, MDA-MB-231-shCtrl cells or MDA-MB-231-shDNMT1 cells were injected into the tail veins of BALB/c female nude mice. We detected breast cancer cell lung metastasis ability by bioluminescence imaging after 8 weeks. The results revealed that the number of MDA-MB-231-shCtrl cells that metastasized to the lung tissues of nude mice was much higher than that of MDA-MB-231-shDNMT1 cells (Fig. 3D, E). Strikingly, histological examination showed that the mice bearing MDA-MB-231-shCtrl cells had a large number of macroscopic lung metastases compared with mice bearing MDA-MB-231-shDNMT1 cells (Fig. 3F, G). Subsequently, we found much larger and much more metastatic foci in the lung tissue sections from mice injected with MDA-MB-231-Ctrl cells than in mice injected with MDA-MB-231-shDNMT1 cells by haematoxylin and eosin (H&E) staining assays (Fig. 3H). Finally, we examined the DNMT1 expression level in lung tissue sections by immunohistochemistry (IHC) assays. We demonstrated that DNMT1 expression in mice injected with MDA-MB-231-shDNMT1 cells was decreased (Fig. 3I). This means that depletion of DNMT1 suppresses breast cancer cell metastasis in vivo. Taken together, our above in vivo data indicate that DNMT1 plays a pivotal role in breast cancer tumorigenesis and distant metastasis.

### ZEB1 regulates DNMT1 transcriptional expression

We have clearly demonstrated that DNMT1 is required for breast cancer cell proliferation and migration in vitro and in vivo. We next intended to illustrate the detailed mechanism of high DNMT1 expression in breast cancer. First, we analysed the DNMT1 promoter sequence by bioinformatics software. The results showed that there were several transcription factor ZEB1 binding motifs (CAGGTG/CACCTG/CACCT). ZEB1 has a great effect on the progression of many cancers. We speculated that whether ZEB1 can regulate DNMT1 expression.

**Fig. 4:**
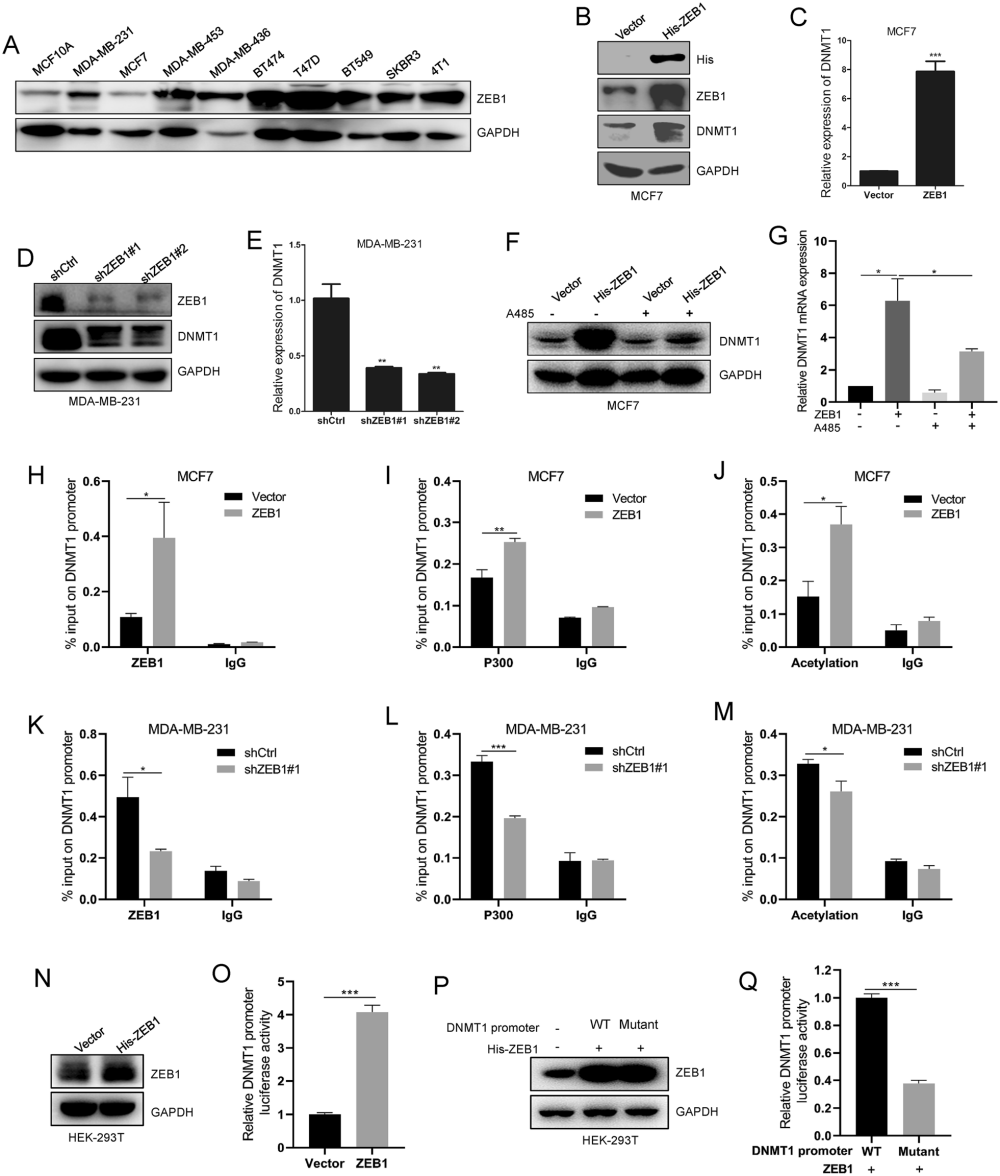
ZEB1 stimulates DNMT1 transcription via increasing its promoter region histone acetylation. **A** Detection of ZEB1 expression in breast cancer cell lines and MCF10A mammary epithelial cell line. **B**, **C** Analysis of DNMT1 expression by western blot (**B**) and qRT-PCR (**C**) in MCF7-Vector cells and MCF7-ZEB1 cells. **D**, **E** Assessment of DNMT1 expression by western blot (**D**) and qRT-PCR (**E**) after silencing DNMT1 in MDA-MB-231 cells. **F**, **G** Detection of DNMT1 expression by western blot (**F**) and qRT-PCR (**G**) after treated with P300 specific inhibitor (60 nM) in MCF7-Vector cells and MCF7-ZEB1 cells. **H**–**J** ChIP assays on *DNMT1* promoter were done using anti-ZEB1, anti-P300 and anti-Acetylation antibodies in MCF7-Vector and MCF7-ZEB1 cells. **K–M** Detection the enrichment of ZEB1, P300 and histones acetylation on *DNMT1* promoter by ChIP assays in MDA-MB-231-shCtrl cells and MDA-MB-231-shZEB1 cells. **N–Q** Luciferase reporter assays detected the DNMT1 promoter region activity after ectopic expression of DNMT1 (**K**) or treated by 500 nM DAC (**L**) in HEK-293T cells; DNMT1 expression was detected by immunoblot assay in HEK-293T-Vector and HEK-293T-DNMT1 cells (**J**). Data are represented as mean ± SD of three independent experiments, and *p < 0.05, **p < 0.01, ***p < 0.001 (Student’s *t* test)

Subsequently, we found that ZEB1 was highly expressed in various breast cancer cell lines (Fig. 4A). Ectopic expression of ZEB1 enhanced DNMT1 expression at the mRNA and protein levels (Fig. 4B, C). In contrast, knockdown of ZEB1 repressed DNMT1 expression at both the transcriptional and protein levels (Fig. 4D, E). Our data suggest that DNMT1 transcription might be activated by ZEB1. Usually, ZEB1 facilitates cancer progression by inhibiting its target gene transcription. Interestingly, a few studies have reported that ZEB1 binding with P300 can increase target gene transcription by increasing target gene promoter histone acetylation [21, 22]. Therefore, we wondered whether ZEB1 stimulates DNMT1 transcription by interacting with P300 and resulting in ZEB1-P300 enrichment on the DNMT1 promoter, which leads to DNMT1 promoter histone acetylation and increased transcription.

To verify our hypothesis, we treated MCF7-Vector cells and MCF7-ZEB1 cells with the P300-specific inhibitor A485 to detect changes in DNMT1 expression. Our data showed that the P300 inhibitor inhibited DNMT1 expression. At the same time, our results revealed that the P300 inhibitor modestly suppressed the ZEB1-induced DNMT1 transcription increase (Fig. 4F, G). Furthermore, we carried out ChIP experiments to detect the enrichment of ZEB1, P300 and histone acetylation at the DNMT1 promoter region in MCF7 cells and MDA-MB-231 cells. On the one hand, we demonstrated that ectopic expression of ZEB1 resulted in much more ZEB1 and P300 binding to the DNMT1 promoter in MCF7 cells (Fig. 4H, I). More histone acetylation was enriched at the DNMT1 promoter in MCF7-ZEB1 cells (Fig. 4J). On the other hand, our ChIP data revealed that the amount of ZEB1, P300 and histone acetylation enrichment at the DNMT1 promoter was decreased after silencing DNMT1 in MDA-MB-231 cells (Fig. 4K–M). Finally, we detected ZEB1 regulating DNMT1 transcriptional activity by luciferase reporter experiments. Our results confirmed that ZEB1 can stimulate DNMT1 promoter activity (Fig. 4N, O). While, ZEB1-mediated DNMT1 promoter activity was strongly attenuated when the ZEB1 binding motif sites on DNMT1 promoter region was mutated (Fig. 4P, Q; and Additional file 1: Fig. S1). Together, our data indicate that ZEB1 activates DNMT1 transcriptional expression, probably through the ZEB1-P300 interaction, and recruits P300 binding to the DNMT1 promoter region, strengthening its histone acetylation.

### TAMs increase DNMT1 expression through the IL-6-pSTAT3-ZEB1-DNMT1 axis

The tumour microenvironment (TME) is very important for cancer cell proliferation and migration. Tumour-associated macrophages (TAMs), as a major kind of immune cell in the TME, have a dramatic effect on cancer progression [23]. A great deal of research has demonstrated that TAMs can accelerate cancer cell migration and metastasis [10, 11, 23]. For instance, TAMs enhance PDPK1-mediated PGK1-T243 phosphorylation in tumour cells by secreting interleukin-6 (IL-6), which enhances TAM-induced tumorigenesis in human glioblastoma multiforme (GBM) [24]; TAM-derived IL-6 activates the JAK2/STAT3 pathway, and the activated STAT3 transcriptionally inhibits the tumour suppressor miR-506-3p in colorectal cancer cells [25]. We speculated that DNMT1 might also play a critical role in TAM-induced breast cancer cell migration.

**Fig. 5:**
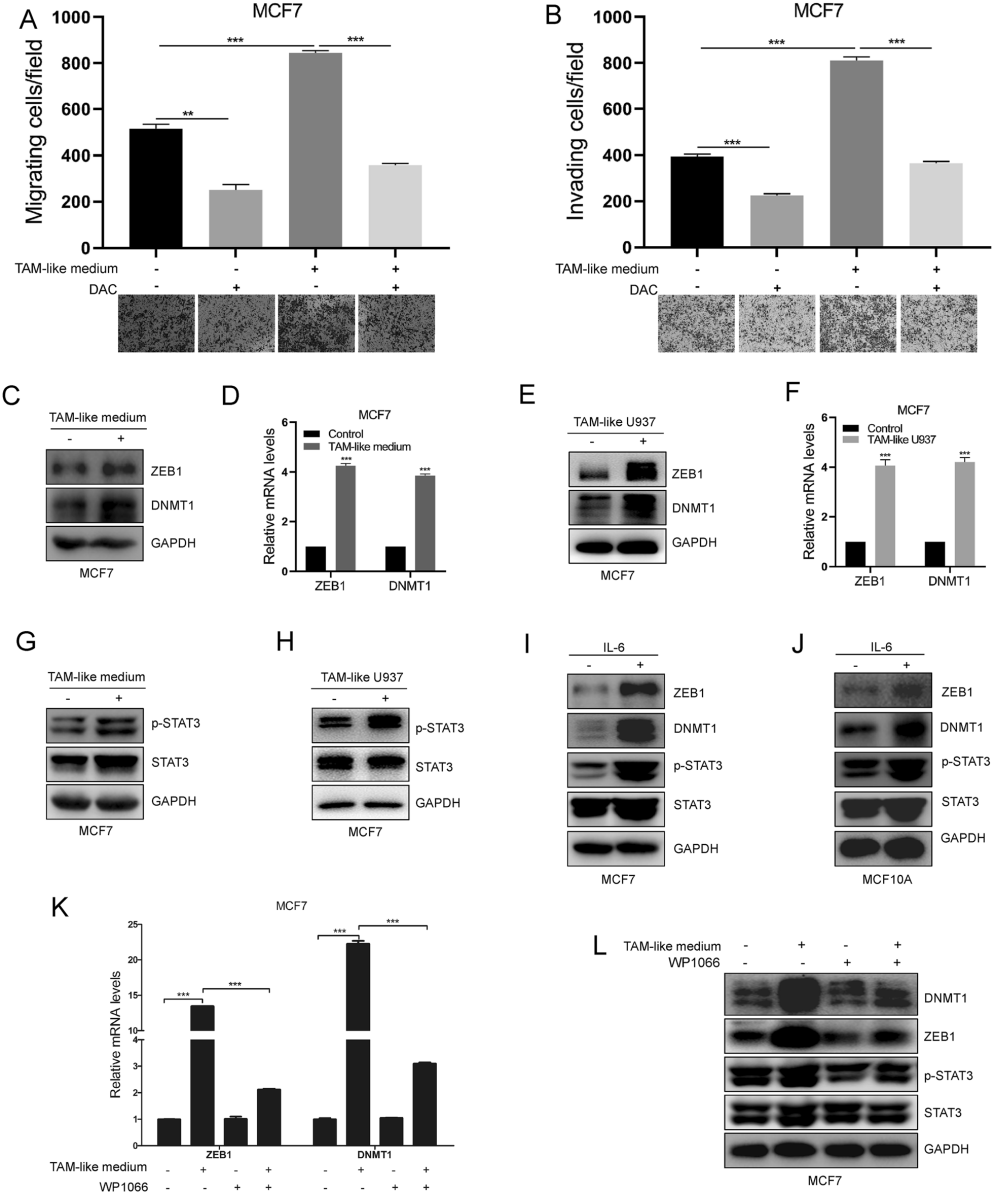
TAMs promotes DNMT1 expression and cell motility in breast cancer cells through IL-6-pSTAT3-ZEB1-DNMT1 axis. **A**, **B** Assessment of cells motility by invasion assays (**A**) and migration assays (**B**) in MCF7-Vector/ZEB1 cells after treated with DMSO or P300 specific inhibitor A485 (60 nM). **C–F** ZEB1 and DNMT1 expression were analyzed by western blot assays (**C**, **E**) and qRT-PCR assays (**D**, **F**) after MCF7 cells were co-cultured with TAM-like medium or TAM-like U937 cells. **G**, **H** STAT3 and p-STAT3 expression were assessed by western blots in MCF7 cells after co-cultured with TAM-like medium or TAM-like U937 cells. **I**, **J** Western blots detection of ZEB1, DNMT1, p-STAT3 and STAT3 expression in MCF10A and MCF7 cells after treated with IL-6 (10 ng/mL, 48 h). **K**, **L** Assessment of ZEB1 and DNMT1 expression by qRT-PCR and western blot in MCF7 cells co-cultured with TAM-like medium after treated with STAT3 inhibitor WP1066 (2.43 μM). Data are represented as mean ± SD of three independent experiments, and *p < 0.05, **p < 0.01, ***p < 0.001 (Student’s *t* test)

To test our hypothesis, we induced monocyte U937 cells into TAM-like cells according to the protocol in previous studies [26, 27]. Strikingly, we found that MCF7 cell invasion and migration abilities were elevated after culture with TAM-like medium or U937-induced TAM-like cells (Fig. 5A, B). However, the increased motility induced by co-cultured with TAM-like medium or U937-induced TAM-like cells in MCF7 cells was restricted by the DNMT1 inhibitor DAC (Fig. 5A, B). This means that DNMT1 is required for TAMs to promote breast cancer cell migration. Interestingly, we found that both ZEB1 and DNMT1 expression at the mRNA and protein levels were increased in MCF7 cells after co-cultured with U937-induced TAM-like cell medium (TAM-like medium) or U937-induced TAM-like cells (Fig. 5C–F). Our data suggest that TAMs can activate ZEB1 and DNMT1 expression.

Next, we determined the detailed mechanism by which TAMs promote ZEB1 and DNMT1 expression. We found that p-STAT3 expression was increased after MCF7 cells were co-cultured with TAM-like medium or U937-induced TAM-like cells. Previous studies have reported that TAM-secreted IL-6 can activate pSTAT3 expression and the STAT3 pathway (Fig. 5G, H). Previous reports have shown that TAM-secreted cytokines play a critical role in TAM-mediated regulation of gene expression. Our recent study also demonstrated that U937-induced TAM-like cells can secrete IL-6 into MCF7 co-cultured medium [14]. We speculated that TAMs enhanced ZEB1-DNMT1 expression through the IL-6-pSTAT3-ZEB1-DNMT1 axis.

Subsequently, our data showed that p-STAT3, ZEB1 and DNMT1 expression was significantly elevated after treatment with IL-6 in MCF7 cells and MCF10A cells (Fig. 5I, J). Finally, we detected ZEB1 and DNMT1 expression after treatment with the STAT3-specific inhibitor WP1066 when MCF7 cells were co-cultured with TAM-like medium. Our results revealed that the elevated expression of ZEB1 and DNMT1 by TAM-like medium at the mRNA and protein levels was dramatically repressed when MCF7 cells were co-cultured with TAM-like medium and treated with the STAT3-specific inhibitor WP1066 (Fig. 5K, L). Overall, our data show that DNMT1 is necessary for TAM-induced breast cancer cell migration in the TME. TAMs promote DNMT1 expression in breast cancer cells via the IL-6-pSTAT3-ZEB1-DNMT1 axis in the TME.

### TAM infiltration positively correlates with ZEB1 and DNMT1 expression in breast cancer tissues

**Fig. 6:**
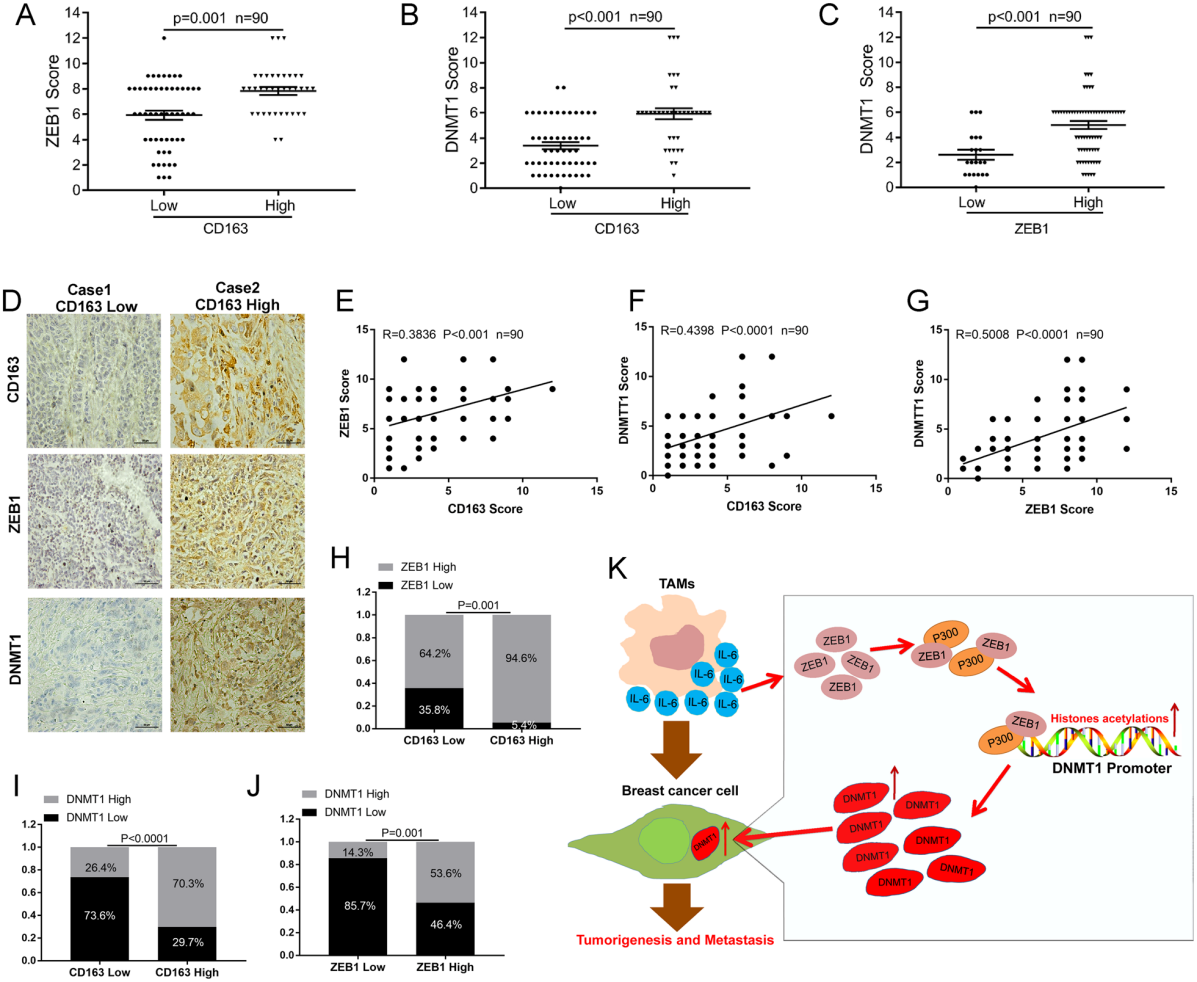
TAMs infiltration positively correlates with ZEB1 and DNMT1 expression in breast cancer tissues. **A–C** IHC assays in breast cancer tissues were measured using anti-CD163, anti-ZEB1 and anti-DNMT1 antibodies (n = 90). Semi-quantitative scoring method (using a scale from 0 to 12) was used to quantify the scores of CD163, ZEB1 and DNMT1 IHC staining. Analyzing the relevant ZEB1 (**A**) or DNMT1 (**B**) expression in CD163-Low cases and CD163-High cases; analyzing the relevant DNMT1 expression in ZEB1-Low cases and ZEB1-High cases (**C**) by Student’s t-test. **D-G** Representative images of CD163, ZEB1 and DNMT1 expressions in CD163-Low case and CD163-High case were presented (**D**). Correlation between CD163 and ZEB1 expression (**E**), CD163 and DNMT1 expression (**F**), ZEB1 and DNMT1 (**G**) were examined by Pearson correlation coefficient test, respectively. **H–J** Correlation between CD163 and ZEB1 expression (**H**), CD163 and DNMT1 expression (**I**), ZEB1 and DNMT1 expression (**J**) were examined by Fisher’s exact test, respectively. **K** A proposed model of this study. TAMs secrete IL-6 to increase breast cancer cells ZEB1 expression through stimulating STAT3 pathway in tumour microenvironment. Then ZEB1 facilitates DNMT1 transcription by recruited P300 enrichment on DNMT1 promoter. Finally DNMT1 high expression promotes breast cancer tumorigenesis and metastasis

Finally, to evaluate the clinical significance of our findings, we performed immunohistochemistry (IHC) staining of breast cancer tissue microarray slides (TMAs) using anti-CD163 (TAM marker), anti-ZEB1 and anti-DNMT1 antibodies. The IHC data revealed that the ZEB1 and DNMT1 expression levels were much higher in breast cancer tissues with high CD163 expression (Fig. 6A, B). Our results also demonstrated that DNMT1 was expressed at high levels in breast cancer patients with high ZEB1 expression (Fig. 6C). Furthermore, we analysed the correlation among CD163, ZEB1 and DNMT1 expression (Fig. 6D–G). Our results revealed that CD163-ZEB1 expression (Fig. 6E), CD163-DNMT1 expression (Fig. 6F) and ZEB1-DNMT1 expression (Fig. 6G) were positively correlated in breast cancer patients. In addition, our data showed that high CD163 expression was positively correlated with high ZEB1 expression (Fig. 6H) and high DNMT1 expression (Fig. 6I). High ZEB1 expression was also positively correlated with high DNMT1 expression in our detected TMA breast cancer tissues (Fig. 6J). These data strongly confirmed our hypothesis that TAM infiltration stimulates DNMT1 expression and promotes breast cancer progression via the IL-6-pSTAT3-ZEB1-DNMT1 axis. Similar to our proposed model, the results are shown in Fig. 6K.

## Discussion

Epigenetic modifications have long been reported as hallmarks of tumour growth and tumour metastasis [28]. DNA methylation, as a critical kind of epigenetic modification, is involved in multiple biological functions, including proliferation, autophagy, apoptosis and immunity, by repressing gene transcriptional expression [29]. The DNA methyltransferase (DNMT) family includes DNMT1, DNMT3a, and DNMT3b in mammalian cells [1]. In this study, we demonstrate that DNMT1 can promote breast cancer cell proliferation and tumorigenesis in vitro and in vivo. DNMT1 can induce the EMT program and facilitate cell motility in breast cancer. Combined with other studies on DNMT1 functions in cancer, these findings strongly indicate that DNMT1 is a core driver gene in tumour progression. Inhibiting DNMT1 expression or its activity is a powerful method for breast cancer therapy.

Many studies have indicated that TAMs facilitate cancer cell migration and metastasis in vitro and in vivo [23]. Nevertheless, the detailed mechanism is not very clear. Whether DNMT1 plays a role in TAM-stimulated breast cancer metastasis remains unclear. Here, we first indicated that DNMT1 is required for TAMs to stimulate breast cancer cell migration. Decitabine (DAC), an inhibitor of DNA methyltransferase, was approved by the FDA for treating haematologic diseases many years ago, such as MDS and CMML [8, 9]. We found that decitabine (DAC) is able to restrict TAM-mediated breast cancer cell migration in vitro. This means that DAC is a promising drug for breast cancer treatment, especially for preventing breast cancer distant metastasis therapy.

ZEB1, as a transcription factor, can participate in cancer progression, especially in EMT progression. Generally, ZEB1 regulates the expression of a variety of genes by repressing their transcription. However, we show that ZEB1 can increase DNMT1 transcription by binding with P300, which is similar to two other recent findings [21, 22]. We believe that ZEB1 activating target gene expression might play a critical role in cancer progression and other biological functions. We should explore these unknown ZEB1-activated target genes in the future.

## Conclusions

Our findings suggest that the IL-6-pSTAT3-ZEB1-DNMT1 axis plays a key role in TAM-induced breast cancer growth and metastasis. Decitabine (DAC) is a bona fide drug for repressing breast cancer cell proliferation and migration. These findings also indicate that the ZEB1-P300-DNMT1 cascade is a potential anticancer therapy pathway for breast cancer.

## Methods

### Cell culture and decitabine and A548 treatment

MCF10A, MCF7, BT549, MDA-MB-231, MDA-MB-453, MDA-MB-436, BT474, T47D, SKBR3 and 4T1 cell lines were purchased from American Type Culture Collection or Cell library of the Chinese Academy of Sciences. These breast cancer cells were cultured as described before [30]. U937 cell line was purchased from American Type Culture Collection. It was cultured with 1640 medium with 10% FBS. For the study of decitabine function, the MDA-MB-231 cells were treated with decitabine for 48 h with 250 nM and 500 nM, respectively. For the study of P300 inhibitor function, the MCF7 cells were treated with A548 (P300 inhibitor) for 48 h with 60 nM.

### Lentivirus infection

We got the shCtrl, shDNMT1#1, shDNMT1#2, Vector, DNMT1 and His-ZEB1 lentiviruses according to the previous protocol using in our laboratory [31]. The shCtrl and shDNMT1 targeting sequences are shown in Additional file 1.

### Cell proliferation assays

4000 Cells were seeded in 96 well plates in triplicate and were performed according to the Cell Counting Kit 8 manufacture’s protocol. For inhibitor treatment, 4000 cells were plated with 500 nM concentration of decitabine in CCK-8 assay.

### Invasion and migration assays

The invasion and migration assays were performed by our previous protocol [30].

### Western blot, Immunoprecipitation (IP) and Co-immunoprecipitation (Co-IP) assays

Western blot, IP and Co-IP experiments were carried out as described previously [32]. The details of antibodies are shown in Additional file 1.

### RNA extraction, reverse transcription and qRT-PCR, and Chromatin immunoprecipitation (ChIP)

These relevant experiments were carried out according to the protocol practically used in our laboratory [30]. ChIP assays were performed using the ChIP Assay Kit (Beyotime, Cat#P2078) according to the manufacturer’s protocol. The primers used to amplify the DNMT1 promoters are shown in the Additional file 1.

### Luciferase reporter assay

The experiments were performed as has been described in our previous study [33]. The pGL3.0-DNMT1 (-1000–0 bp) reporter gene plasmids (DNMT1 promoter WT and Mutant) were constructed by Biogot technology Company. The detailed protocol and the DNMT1 promoter WT and Mutant DNA sequences (-1000–0 bp) are shown in Additional file 1.

### Animal works of tumour xenograft model and lung-colonization metastasis model

The animal experiments were approved by the Animal Care Committee of Xuzhou Medical University, Xuzhou, China. Female BALB/c nude mice (6–8 weeks old) were obtained from the GemPharmatech Company. The tumour xenograft model and the lung-colonization metastasis model procedures were performed as previously described [34].

### Breast cancer tissues and immunohistochemistry (IHC) assay

The breast cancer tissue microarrays (TMAs) for staining CD163, ZEB1 and DNMT1 were purchased from Bioaitech Company (containing 90 breast cancer tissue specimens). IHC assays were carried out using a standard streptavidin-Peroxidase method in our previous study [35]. The detailed method of IHC assessment and the primary antibodies were described in the Additional file 1.

### Statistical analyses

All data are presented as mean ± SD at least three biological experiments. Statistical analyses of prostate cancer tissues staining were performed with SPSS 20 software. The Student's *t* test was used to analyze statistic significance of differences among two groups. *p* < 0.05 was considered statistically significant. Statistical analyses were carried out by using the GraphPad Prism software.

## Supplementary Information

**Additional file 1: Fig. S1.** The DNMT1 promoter WT and mutant DNA sequences

## Data Availability

The datasets used and/or analyzed during the current study are available from the corresponding author on reasonable request.

## Abbreviations

EMT: Epithelial-Mesenchymal Transition
TAMs: Tumour-associated macrophages
TME: Tumour microenvironment
DAC: Decitabine
IHC: Immunohistochemistry
GBM: Glioblastoma multiforme
DNMT: DNA methyltransferase
