# Supplementary material for: Tumour-associated macrophages enhance breast cancer malignancy via inducing ZEB1-mediated DNMT1 transcriptional activation
Source: Cell Biosci. 2022 Oct 22;12:176. doi: 10.1186/s13578-022-00913-4 (PMC9587673; doi:10.1186/s13578-022-00913-4)

**Tumour-associated macrophages enhance breast cancer malignancy via inducing ZEB1-mediated DNMT1 transcriptional activation**

Zhongwei Li^1,2,3#^, Pengfei Wang^1#^, Wenjie Cui^4#^, Hongmei Yong^1^, Diandian Wang^5^, Tiesuo Zhao^6^, Wenwen Wang^1^, Ming Shi^1,2,3*^, Junnian Zheng^2,3*^ and Jin Bai^1,2,3*^

**Affiliations**

^1^Cancer Institute, Xuzhou Medical University, Xuzhou, Jiangsu, China.

^2^Center of Clinical Oncology, the Affiliated Hospital of Xuzhou Medical University, Xuzhou, Jiangsu, China.

^3^Jiangsu Center for the Collaboration and Innovation of Cancer Biotherapy, Cancer Institute, Xuzhou Medical University, Xuzhou, Jiangsu, China.

^4^Department of Respiratory and Critical Care Medicine, The Municipal Hospital Affiliated to Xuzhou Medical University, Xuzhou, Jiangsu, China.

^5^Intensive Care Unit, The Second Affiliated Hospital of Xuzhou Medical University, Xuzhou, Jiangsu, China.

^6^Department of Immunology, School of Basic Medical Sciences, Xinxiang Medical University, Xinxiang, Henan, China.

^#^These authors contributed equally to this paper.

**Correspondence Authors:**

Jin Bai, Cancer Institute, Xuzhou Medical University. 84 West Huaihai Road, Xuzhou, 221002, Jiangsu Province, China. E-mail: bj@xzhmu.edu.cn.

Junnian Zheng, Cancer Institute, Xuzhou Medical University. 84 West Huaihai Road, Xuzhou, 221002, Jiangsu Province, China. E-mail: jnzheng@xzhmu.edu.cn.

Ming Shi, Cancer Institute, Xuzhou Medical University. 84 West Huaihai Road, Xuzhou, 221002, Jiangsu Province, China. E-mail: sm200@sohu.com.

**Running title:** TAMs promotes cancer progression by increasing DNMT1 expression

**Keywords:** DNMT1; ZEB1; Tumour-associated macrophages; Metastasis; Breast Cancer

**Conflict of interest:** The authors declare no conflict of interest.

**1 shRNA, qRT-PCR primer and ChIP assay primer sequences**

shCtrl targeting sequence: TTCTCCGAACGTGTCACGT

shDNMT1#1 targeting sequence: GGAATGGCAGATGCCAACAGC

shDNMT1#2 targeting sequence: CGGTGCTCATGCTTACAAC

GAPDH qRT-PCR Forward primer: ATGACCCCTTCATTGACCTCA

GAPDH qRT-PCR Reverse primer: GAGATGATGACCCTTTTGGCT

Ecadherin qRT-PCR Forward primer: GACAACAAGCCCGAATT

Ecadherin qRT-PCR Reverse primer: GGAAACTCTCTCGGTCCA

N-cadherin qRT-PCR Forward primer: CGGGTAATCCTCCCAAATCA

N-cadherin qRT-PCR Reverse primer: CTTTATCCCGGCGTTTCATC

Vimentin qRT-PCR Forward primer: GAGAACTTTGCCGTTGAAGC

Vimentin qRT-PCR Reverse primer: GCTTCCTGTAGGTGGCAATC

Fibronectin qRT-PCR Forward primer: CAGTGGGAGACCTCGAGAAG

Fibronectin qRT-PCR Reverse primer: TCCCTCGGAACATCAGAAAC

ZEB1 qRT-PCR Forward primer: TGCACTGAGTGTGGAAAAGC

ZEB1 qRT-PCR Reverse primer: TGGTGATGCTGAAAGAGACG

DNMT1 ChIP Forward primer: CCGTAGTGACCCACCTAA

DNMT1 ChIP Reverse primer: GGGATAAAGCAGCGAGAA

**2 Reagents**

| **REAGENT or RESOURCE** | **SOURCE** | **IDENTIFIER** |
| --- | --- | --- |
| Decitabine | Selleckchem | Cat#S1200 |
| A485 | Selleckchem | Cat#S8740 |
| IL-6 | MedChemExpress | Cat#HY-P7044 |
| WP1066 | Selleckchem | Cat# S2796 |
| DMEM | Sigma-Aldrich | Cat#D7777 |
| RPMI-1640 | Sigma-Aldrich | Cat#R7755 |
| Fetal Bovine Serum | TRANSGEN BIOTECH | Cat#FS201-02 |
| anti-Acetylation | Cell Signaling Technology | Cat#9441S |
| anti-DNMT1 | Cell Signaling Technology | Cat#5032S |
| anti-ZEB1 | Cell Signaling Technology | Cat#3396S |
| anti-GAPDH | Proteintech | Cat#60004-1 |
| anti-STAT3 | Cell Signaling Technology | Cat#4904S |
| anti-pSTAT3 | Cell Signaling Technology | Cat#9145S |
| anti-CD163 | Cell Signaling Technology | Cat#93498S |
| anti-Cyclin-D1 | Cell Signaling Technology | Cat#2978T |
| anti-Cyclin-E2 | Cell Signaling Technology | Cat#4132T |
| anti-Cyclin-B1 | Cell Signaling Technology | Cat#12231T |
| anti-Cyclin-A2 | Cell Signaling Technology | Cat#67955T |
| anti-Ki67 | GeneTeX | Cat#GTX20833 |

**3 Method of IHC assessment**

CD163, DNMT1 and ZEB1 staining were evaluated blindly and independently by different pathologists. The signals were quantified according to both the intensity and percentage of cells with positive staining. The CD163, DNMT1 and ZEB1 staining intensity was scored 0 to 3 (0 = negative; 1 = weak; 2 = moderate; 3 = strong). The percentage of positive stained cells was also scored into four categories: 1 (0%-25%), 2 (26%-50%), 3 (51%-75%) and 4 (76%-100%). The IHC Score level of CD163, DNMT1 and ZEB1 staining was evaluated by IRS, which is calculated by multiplying the scores of staining intensity and the percentage of positive cells. Based on IRS, the CD163, DNMT1 and ZEB1 staining pattern was categorized as negative (IRS: 0), weak (IRS: 1-3), moderate (IRS: 4-6) and strong (IRS: 8-12).

**4 Method of Luciferase reporter assay**

The DNMT1 promoter luciferase plasmid (WT or Mutant) was transiently transfected into HEK293T cells; at the same time Renilla plasmid (Promega) and His-ZEB1 or Vector expression plasmid were co-transfected. Forty-eight hours later, cells were harvested, and luciferase activities were measured by the Dual-Luciferase Reporter Assay Kit (Promega) on a luminometer (Molecular Devices, Sunnyvale, CA, USA). And DNMT1 promoter relative firefly luciferase activity was normalized to the Renilla activity.

**Supplementary Figure Legend**

**Fig. S1.** The DNMT1 promoter WT and mutant DNA sequences.


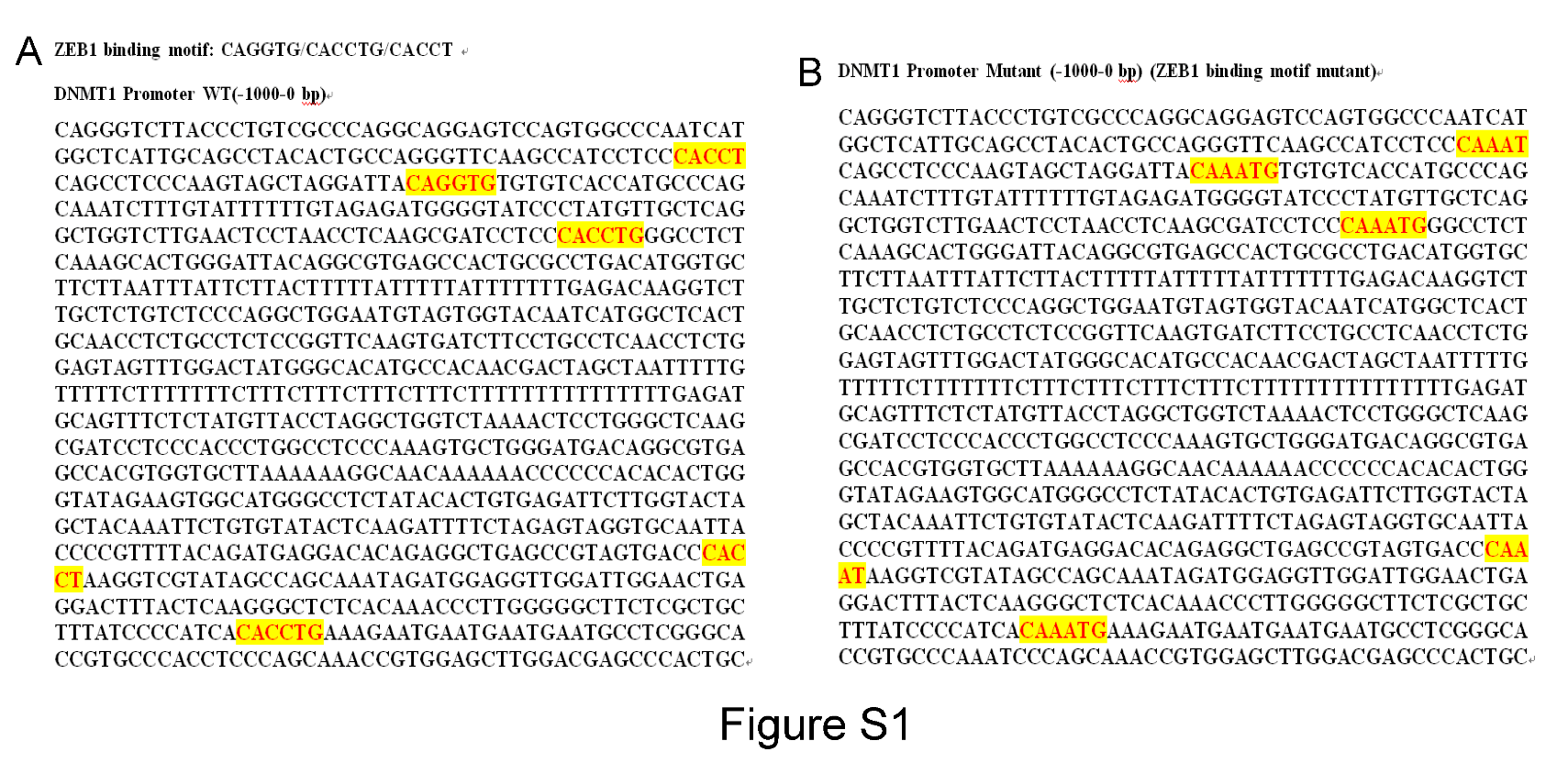

Supplement: Supplementary file 1 — Additional file 1: Fig. S1. The DNMT1 promoter WT and mutant DNA sequences [file 13578_2022_913_MOESM1_ESM.docx]
